# Investigating Statistical Conditions of Coevolutionary Signals that Enable Algorithmic Predictions of Protein Partners

**DOI:** 10.1021/acs.jcim.5c00052

**Published:** 2025-04-15

**Authors:** José Fiorote, João Alves, Letícia Stock, Werner Treptow

**Affiliations:** 1 Laboratório de Biologia Teórica e Computacional (LBTC), Universidade de Brasília, Brasilia, DF 70910-900, Brasil; 2 Ben May Department for Cancer Research, University of Chicago, Chicago, Illinois 60637, United States

## Abstract

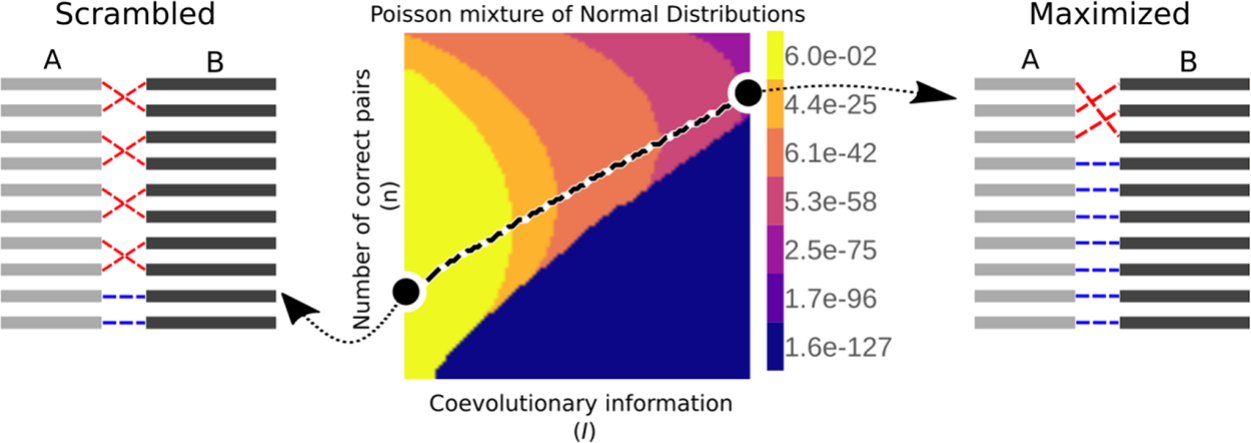

This study examines the statistical conditions of coevolutionary signals that allow algorithmic predictions of protein partners based on amino acid sequences rather than 3D structures. It introduces a Markov stochastic model that predicts the number of correct protein partners based on coevolutionary information. The model defines state probabilities using a Poisson mixture of normal distributions, with key parameters including the total number of protein sequences *M*, the coevolutionary information gap α, and variance σ_0_^2^. The model suggests that algorithmic approaches that maximize coevolutionary information cannot effectively resolve partners in protein families with a large number of sequences *M* ≥ 100. The model shows that true-positive (TP) rates can be enhanced by disregarding mismatches among similar sequences. This approach allows a distinction, in terms of {α, σ_0_^2^}, between optimized solutions with trivial errors and other degenerate solutions. Our findings enable the a priori classification of protein families where partners can be reliably predicted by ignoring trivial errors between similar sequences, advancing the understanding of coevolutionary models for large protein data sets.

Amino acid contacts between proteins *A* and *B* are maintained over the course of evolution through compensatory mutations.^1^ Over the years, extensive investigations have highlighted the relevance of coevolutionary signals for *ab initio* inference of protein partners based on primary sequence analysis.^2,3^ Simulations optimizing various estimators of the coevolutionary signal, such as mutual information (I),^4−7^ direct information (DI),^7,8^ or mirror tree (R),^7,9^ have been proved useful in resolving partners *A* and *B* within paralogous families containing a small number of protein copies per genome. However, the predictive challenge remains unresolved for the general case of interaction systems because the latter estimators are an ineffective heuristic when the number of proteins increases. In families containing tens to hundreds of proteins, simulations starting from random partner assignments for *A* and *B* consistently fail to correctly pair them, even after optimizing the coevolutionary information.^6^

The low true-positive (TP) rates observed in the optimization procedures are likely due to a significant degeneracy in the coevolutionary signal across the space of possible matches between proteins *A* and *B*. Despite this intuition, the lack of a quantitative understanding of the problem prevents further progress in the field. In one recent advance, predictions of protein partners at the level of coevolutionary clades have been shown to be potentially achievable by disregarding trivial errors made among similar sequences.^6^ Optimized solutions that tolerate such trivial errors have since then been proposed to hold significant promise for predictive purposes; however, their practical realization depends on the ability to statistically distinguish these solutions from other degenerate ones. Consequently, achieving this distinction has emerged as a pivotal aspect of the problem that needs to be addressed.

Here, we present a statistical framework designed to rigorously explore the generation of TP rates in optimization procedures of coevolutionary information. Particular emphasis is placed on analyzing TP rates with or without incorporating the reassessment of trivial errors, providing insight into their impact on the predictive accuracy. The statistical structure consists of a Markov stochastic model of the number of correct protein partners *n* and coevolutionary information *I*, where the state probabilities are defined according to a Poisson mixture of Normal distributions, parametrized by a reduced set of relevant variables {*M*, α, σ_0_^2^}, respectively the total number of protein sequences, the gap of coevolutionary information, and the variance. In our results, we find that the dominant Poisson weight of random pairs *A* and *B* makes them the most likely Markov state across the domain {*n*, *I*}. Starting with the most likely random states, resolving the model via maximization of the coevolutionary information establishes well-defined conditions {*M*, α, and σ_0_^2^} under which TP rates are significant (≥0.5). These conditions are significantly modified by reassessing similar sequences, enabling a clear distinction between optimized solutions with trivial errors and other degenerate alternatives. In agreement with simulated data of various protein families, our quantitative findings recapitulate real system data and allow for an a *priori* classification of optimized solutions. By differentiating solutions with trivial errors, we are able to effectively resolve partners *A* and *B*.

## Theory and Methods

**Scheme 1:**
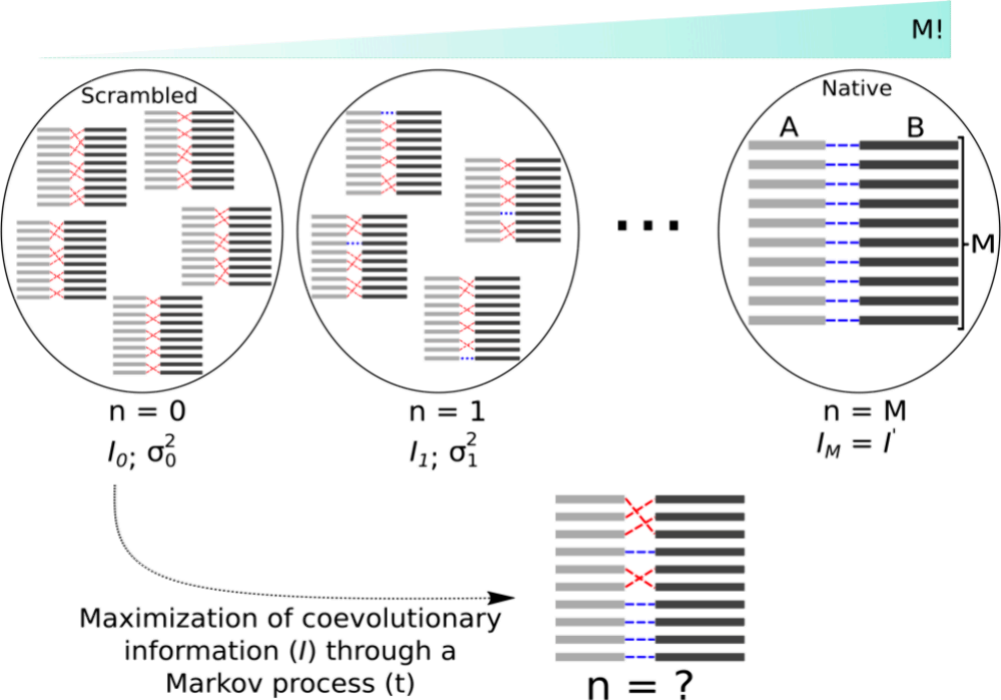
Two Sets, *A* and *B*, Each Consisting of *M* Protein Sequences These proteins can be paired in *M*! distinct ways, with each pairing characterized by the number of correct partners (*n*) and the coevolutionary information content (*I*). The “native” arrangement is defined as the one in which all partners are correct (*n* = *M*) and the information content is maximal (*I*_*M*_ = *I*′). Based on this framework, we address the following problem: Consider a distribution of scrambled arrangements where the number of correct partners between *A* and *B* is *n* = 0. This distribution is characterized by its variance σ_0_^2^ and coevolutionary information *I*_0_. Starting from any arrangement within this scrambled distribution, what is the most likely number of correct partners after maximizing *I*? To tackle this problem, our statistical model assumes that maximization follows a Markov process, with state probabilities described by a Poisson mixture of Normal distributions.

Consider a general finite set of *M* protein sequences *A* and *B*, individually containing *N* amino acids. Proteins *A* and *B* may be paired to each other according to *M*! distinct arrangements *r*, characterized by the number of correct partners 0 ≤ *n*(*r*) ≤ *M* and the coevolutionary information content *I*_0_ ≤ *I*(*r*) ≤ *I*′. The “native” arrangement is assumed to be unique *r*′, with *n*(*r* ′) = *M* and *I*(*r* ′) = *I*′. Together, the joint variables {*n*, *I*} help to define a set of discrete states *c* described by the stochastic variable *C*, with probability mass function *P*(*c*). Time evolution of the stochastic variable *C* is assumed to follow a Markov process, with the transition probability given by *p*_*c*_*t*_,*c*_*t*+1__ = *P*(*c*_*t*+1_ | *c*_*t*_). Under these considerations, we seek to devise a statistical model for the time evolution of stochastic variable *C* subjected to optimization of the coevolutionary information. Special attention is given to modeling optimized trajectories with or without the reassessment of errors made between similar sequences within *A* or *B* (Scheme 1).

### Distribution of Coevolutionary Information and Sequence Matches

In order to investigate the prediction of protein partners along optimized trajectories of the stochastic variable *C*, we start by modeling the statistical distribution of the joint variables {*n*, *I*} according to a mixture model
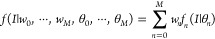
1in which, *f*_*n*_(.) is the distribution of the coevolutionary information with parameters θ_*n*_. *w*_*n*_ ⩾ 0 is the mixing weight relative to the number of correct pairs such that
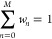
2and
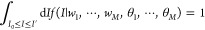
3

The coevolutionary information is a continuous variable resulting from the sum of *N*^2^ – *N* contributions,^10^ and, as such, it is expected to be normally distributed (central limit theorem)
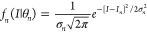
4with θ_*n*_ = {*I*_*n*_, σ_*n*_^2^} denoting the mean and variance of the distribution for a fixed number of correct partners *n*. Because the total number of *rencontres* generated by uniformly distributed random permutations of the native arrangement is well-defined
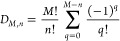
5the mixing weight of each normal distribution *w*_*n*_ converges to the probability mass function of the Poison distribution with expected value λ = 1
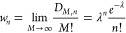
6provided that *M* is sufficiently large and *M* ! = ∑_*n* = 0_^*M*^*D*_*M*, *n*_.

From eqs 4 and 6, the statistical model in eq 1 can be rewritten as a weighted composition of normal distributions, such that the *n*th component of the mixture is given by
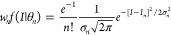
7

### Time Evolution of the Stochastic Variable *C*

The probability density *w*_*n*_*f*_*n*_(*I* | θ_*n*_) is particularly important as it allows for solution of the time evolution of the stochastic variable *C*. More specifically, the Information domain can be discretized into *k* bins of length δ*I*, allowing the probability mass function *P*(*c*) of a given discrete state *c* = {*n*, (*I* – δ*I*/2) ≤ *I* < (*I* + δ*I*/2)} to be determined accordingly. Following the quantification of the transition probabilities *p*_*c*_*t*_,*c*_*t*+1__ along every time step of the optimization process *t*,
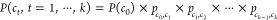
8then writes as the probability of any specific trajectory of the stochastic variable *C* between the initial state *c*_0_ and the absorbent state *c*_*k*_. Of particular interest is the fact that the most likely trajectory {*c*_t_^*^, *t* = 1, ···, *k*} can be determined via maximization of eq 8 across all possible transitions between the states *c*_0_ and *c*_*k*_.

### Reassessment of the Time Evolution of *C* by Disregarding Mismatches Made among Similar Sequences

Careful inspection of the distinct arrangements within each component *n* of the mixture model indicates that an effective true-positive (TP) rate may be obtained by reassessing the number of mismatches between sequences *A* and *B*. Accordingly, the model is reformulated to account for an effective number of protein sequences *n*′ that are paired either with their correct partner or with a similar partner defined according to a Hamming distance cutoff. More specifically, by setting *m* as the number of similar partners, we consider an invariant transformation of the model
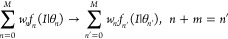
9where every *n*th component of the mixture is expanded
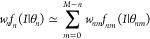
10in terms of the auxiliary distributions *w*_*nm*_*f*_*nm*_(.). The expansion procedure allows the model to be restated as
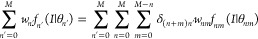
11by the combination of the individual components *w*_*nm*_*f*_*nm*_(.) in which the number of similar partners satisfies the Kronecker delta δ_(*n*+*m*)*n*′_. In the limit of a valid approximation of the distribution parameters θ_*nm*_ ≈ θ_*n*_ for all *n* where *w*_*n*_ > 0, eq 11 simplifies to
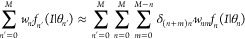
12with
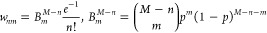
13given by a finite set of Bernoulli processes in which *M*-long arrangements with *n* fixed positions contain 0 ≤ *m* ≤ *M* – *n* similar partners with probability *p*. The implication for the mixture model is that the distribution function in eq 1 transforms into reweighted mixture of normal distributions
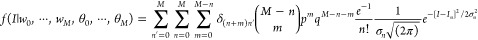
14

### Computational Methods

For any given number of fixed positions 0 ≤ *n* ≤ *M*, θ_*n*_ = {*I*_*n*_, σ_*n*_^2^} was defined from the scrambled parameters θ_0_ = {*I*_0_, σ_0_^2^} as polynomial functions
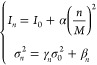
15with
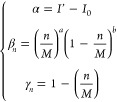
16satisfying the condition {*I*_*M*_ = *I* ′, σ_*M*_^2^ = 0}. Based on the free parameters {*M*, α, σ_0_^2^, *a*, and *b*}, these equations well describe the *n*-dependent behavior of the coevolutionary information and variance of protein systems, therefore justifying our choice (see Results and Discussion).

Serving as input functions, eqs 15 and 16 were used to solve the statistical model in eqs 1–8 across a broad range of conditions. For each set of free parameters, the probability density *w*_*n*_*f*_*n*_(*I* | θ_*n*_) was regularized according to the normalization conditions in eqs 2 and 3. The information domain *I*_0_ ≤ *I* ≤ *I*′ was discretized into *k* = *M* bins of length δ*I* ≤ *M*^–1^(*I* ′ – *I*_0_), and the mass probability *P*(*c*) of each discrete state *c* = {*n*, (*I* – δ*I*/2) ≤ *I* < (*I* + δ*I*/2)} was determined from the difference of the cumulative distribution function of eq 7 across the interval. Transition probabilities were computed as an element of a right stochastic (reducible) matrix *p*_*c*_*t*_,*c*_*t*+1__ = *P*(*c*_*t*+1_)/∑_*c*_*t*+1_^′^_*P*(*c*_*t*+1_^′^), by assuming an optimization process *I*_*t*_ < *I*_*t*+1_ in which two sequences are swapped per time step, such that *n*_*t*+1_ ∈ {*n*_*t*_ – 1, *n*_*t*_, *n*_*t*_ + 1}. The most likely optimized trajectory was solved by maximization of eq 8 across all possible transitions between *c*_0_ and the absorbent state *c*_*k*_. The initial state *c*_0_ was chosen according to a maximum mass probability criterium max[*P*(*c*_0_)]. For each set of the model’s parameters, the true-positive (TP) rate is defined as the fraction of correct partners in the absorbent state of the most likely trajectory.

The statistical model was reassessed according to eqs 9–14, by considering the effective number of sequences *n*′ that are paired either with their correct partner or with a similar partner. Similar partners of sequences *A* were defined based on a Hamming distance cutoff set at the 20th percentile of the distribution of Hamming distances of sequences *B* (*p* = 0.2). The approximation θ_*nm*_ ≈ θ_*n*_ in eq 12 was investigated for small values of *n* for which Poisson weights *w*_*n*_ are relevant, i.e., *w*_*n*_ > 0 and 0 ≤ *n* ≤ 16. Sequence arrangements with a given number of correct fixed positions *n* were randomly generated and subsequently distributed according to the number of similar partners *m*. The average and variance of mutual information θ_*nm*_ in each subset of the distribution were computed and compared to the corresponding estimates from the source distribution θ_*n*_.

## Results and Discussion

Here, we investigate the statistical model in eqs 1–8. Our primary goal is to gain quantitative insights into the true-positive (TP) rate achieved through optimizations of the coevolutionary information in amino acid sequences. The number of correct partners was defined in the Theory and Methods section as a value between 0 ≤ *n* ≤ *M*, where *M* is the total number of protein sequences. Accordingly, the TP rate is defined as .

### Definition of the Input Functions of the Model

Equations 15 and 16 well describe the *n*-dependent behavior of the coevolutionary information and variance of “real” protein families, therefore justifying our choice. Equations 15 and 16 then served as input functions to solve our statistical model across a broad range of numerical conditions.

More specifically, eqs 15 and 16 were mathematically defined to describe the *n*-dependent behavior of the coevolutionary information and variance of protein families shown in Table S1. Each family contains a certain number *M* of protein interologs of types *A* and *B*, with known protein interactions, i.e., with a known native arrangement in the context of our model. Details on the curated multiple sequence alignments (MSAs) for each of these protein families are provided in previous publications. In brief, MSAs for the HK-RR standard data set were generated using the P2CS database^11^ and validated by Bitbol and colleagues.^8^ MSAs for all orthologous protein families were obtained from Ovchinnikov and collaborators,^12^ who used HMMER 3.1^13^ to construct HMM profiles and search for PDB sequences in the S2C database. The paired alignments were filtered to limit redundancy to 90% sequence identity and to remove positions with more than 75% gaps. Because the native arrangement is known in both prokaryotic data sets, they have been commonly used as a benchmark for studying the inference of interaction partners from protein sequences.^6,8^

**Figure 1:**
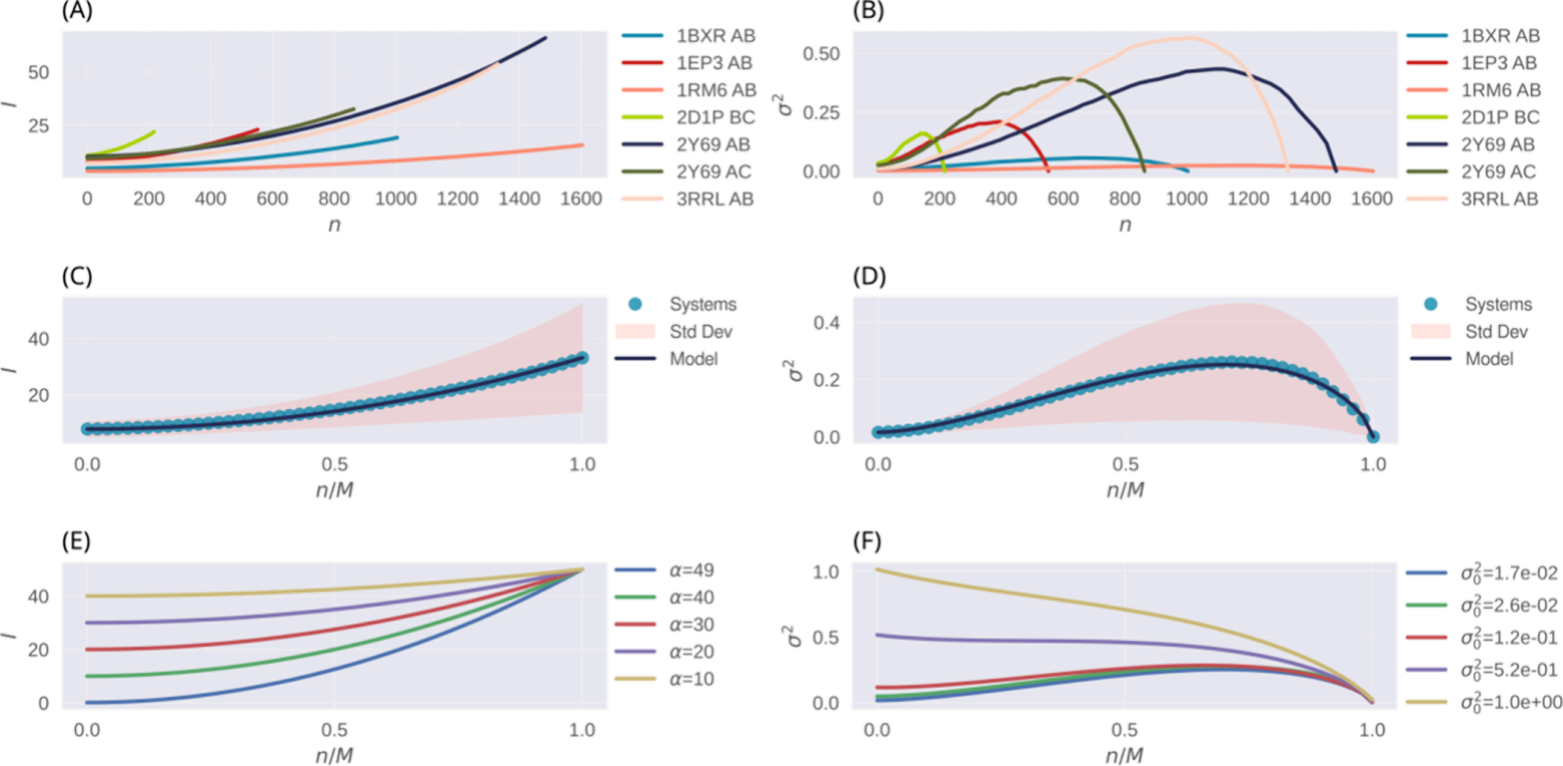
Coevolutionary information and variance σ_*n*_^2^ as a function of the number of correct partners *n*. (A, B) Shown is the coevolutionary information *I*_*n*_ and variance σ_*n*_^2^ of the protein families 1BXR, 1EP3, 1RM6, 2D1P, 2Y69, and 3RRL (see Table-S1 for details). *I*_*n*_ and σ_*n*_^2^ are average estimates determined from ∼10,000 randomly generated arrangements with a fixed number of positions *n*. (C, D) Mean values of *I*_*n*_ and σ_*n*_^2^ (blue points). Mean values were calculated from data in (A), after regularization by the total number of sequences *M*. Black curves are the best fits of the input functions over *I*_*n*_ and σ_*n*_^2^ (eqs 15 and 16). With regression coefficients *R* ≥ 0.99, best-fit parameters are α = 25.93, σ_0_^2^= 0.02, *a* = 1.63, and *b* = 0.68. (E, F) Dependence of the input functions with free parameters {*M*, α, and σ_0_^2^}. As a relative measure of coevolutionary information, α = *I* ′ – *I*_0_ was defined in terms of a fixed information content of the native arrangement, i.e., *I* ′ = 50.

Since the protein pairs are known for each of these protein families, the relationship between coevolutionary information *I*_*n*_ and variance σ_*n*_^2^ with the number of correct partners *n* can be investigated through the scrambling of the native arrangement. For clarity, Figure 1A and Figure 1B respectively illustrate the *n*-dependent behavior of coevolutionary information and variance for a few representative protein families from Table S1. For each protein family, coevolutionary information and variance were numerically determined by averaging over approximately 10,000 randomly generated arrangements, each with a fixed number of positions *n*. The coevolutionary information, measured in *nats*, was computed as described by Andrade et al.^10^ This calculation took into account the Shannon mutual information between amino acids involved in close molecular interactions between proteins *A* and *B*.

Parametrized by {*M*, α, σ_0_^2^, *a*, *b*}, eqs 15 and 16 effectively capture the average behavior of the coevolutionary information and variance of the protein families under consideration (Figure 1C,D). To simplify the investigation of the model’s behavior and focus on fewer relevant variables, the free parameters were restricted to {*M*, α, and σ_0_^2^}, with the remaining degrees of freedom held constant and set to values that best fit the input functions to the data. Since our primary goal was to gain quantitative insights into the TP rate achieved through the optimization of coevolutionary information in amino acid sequences in general, the relevant parameters were selected across a broad range of numerical values. These values included, but were not limited to, typical values for the protein families presented in Table S1. Figure 1E,F illustrates the behavior of the input functions across the range of free parameters evaluated in the study.

### Solution of the Statistical Model

**Figure 2:**
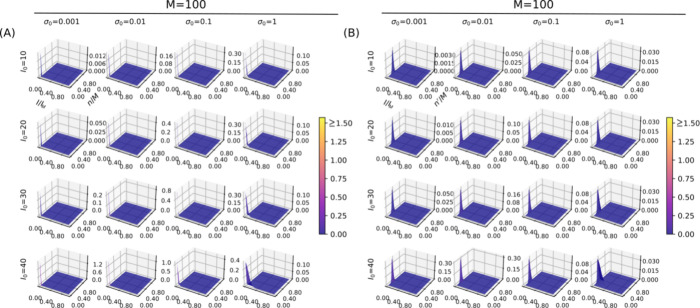
Dependence of the statistical model with {*M*, α, σ_0_^2^}. (A) Weighted probability density of coevolutionary information *w*_*n*_*f*_*n*_(*I* | θ_*n*_) as a function of the number of correct partners *n*. (B) Reassessment of the statistical model *w*_*n*′_*f*_*n*′_(*I* | θ_*n*′_) by disregarding mismatches made among similar sequences.

**Figure 3:**
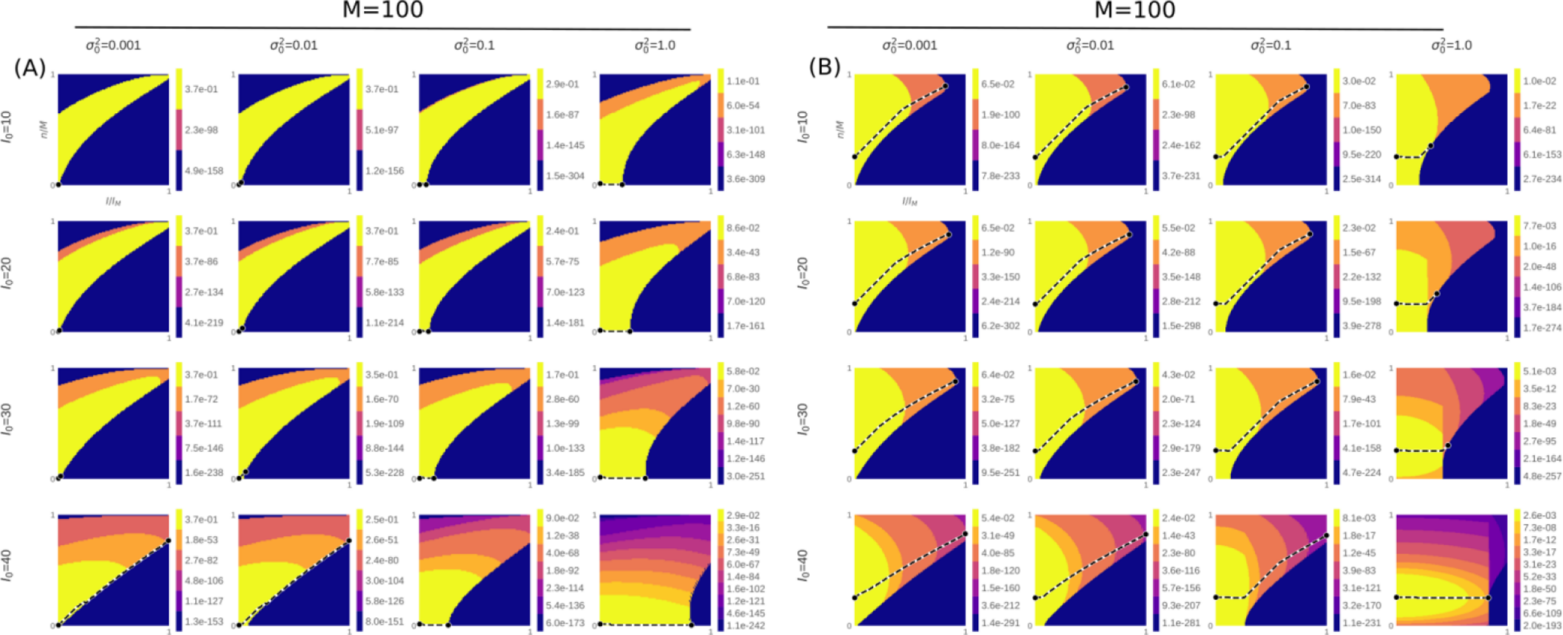
Time evolution of the stochastic variable *C* subjected to optimization of the coevolutionary information. Shown is the mass probability function *P*(*c*) before (A) and after (B) the reassessment of mismatches made among similar sequences. Traces indicate the most likely trajectories {*c*_t_^*^, *t* = 1, ···, *k*} solved by maximization of eq 8 across all possible transitions between *c*_0_ and the absorbent state *c*_*k*_. The initial state *c*_0_ was chosen according to a maximum mass probability criterium max[*P*(*c*_0_)].

The data used to solve the statistical model in eqs 1–8 are derived from the input functions shown in Figure 1E,F. For each specific value of free parameters shown in Figure 1E,F, the weighted probability density of the model is computed, as depicted in Figure 2, and is then applied to resolve the Markov state probabilities, shown in Figure 3. The final outcome of the model is the number of correct partners in the absorbing state of the most likely trajectory, which follows optimization of the coevolutionary information.

In more detail, our framework is based on a Poisson mixture of normal distributions of the number of correct protein partners *n* and coevolutionary information *I*. According to the central limit theorem, the probability density of coevolutionary information *f*_*n*_(*I* | θ_*n*_) is expected to be Gaussian, a prediction supported by Figure S1, which shows the numerically generated distribution of the coevolutionary signal for the 1BXR_AB protein family. Additionally, the Poisson weight *w*_*n*_ shown in Figure S2 is exact for large values of *M* > 100, accurately reflecting the number of arrangements of protein systems as a function of the number of correct partners. These two mathematical properties of the weighted probability density *w*_*n*_*f*_*n*_(*I* | θ_*n*_) serve as the ground truth of the model, establishing it as a robust framework for describing the statistical distribution of joint variables in protein systems composed of a large number of sequences.

Parametrized by *I*_*n*_ and σ_*n*_^2^ in Figure 1E,F, the probability density of the coevolutionary information *f*_*n*_(*I* | θ_*n*_) is a multipeaked distribution over the domain of the joint variables {*n*, *I*} (Figure S3). However, when subjected to Poisson weights *w*_*n*_, the weighted probability density *w*_*n*_*f*_*n*_(*I* | θ_*n*_) transforms into a single-peaked distribution (Figure 2). The density peak of the weighted distribution occurs in the scrambled region of the domain {*n*, *I*} driven by the dominant values of *w*_*n*_ at small *n* (Figure S2). As such, the model successfully captures a key feature of protein systems: scrambled pairs, which exhibit low values for the joint variables {*n*, *I*}, are the most likely configurations across the entire space of possibilities. In other words, scrambled arrangements are readily produced through random permutations of native protein pairs, as demonstrated in Figure 1B of Andrade et al.^10^

**Figure 4:**
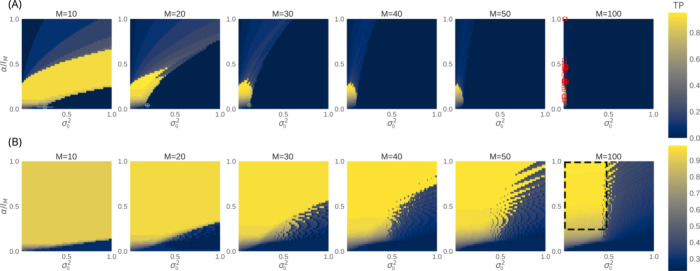
Prediction of TP rates. Shown are the true-positive (TP) rates before (A) and after (B) reassessing mismatches among similar protein partners. As indicated in Table S1, simulated TP rates deteriorate significantly when the parameters of paralogous (circle) and orthologous (square) systems fall outside the model’s sweet spot domain (yellow). Simulated rates are color-coded as red (0.0 ≤ TP < 0.2), green (0.2 ≤ TP ≤ 0.5), and purple (0.5 < TP ≤ 1.0). The model’s predicted region of the parameter space, likely containing optimized solutions with trivial errors, is indicated by the dashed box (see main text for details).

Despite their overall similarity imposed by *w*_*n*_, careful inspection of the weighted distributions in Figure 2 reveals a significant numerical dependence on the choice of the free parameters, as reflected on the optimization trajectories in Figure 3. More specifically, the implications of the weighted density *w*_*n*_*f*_*n*_(*I* | θ_*n*_) on the mass probability function *P*(*c*) are consistent, making scrambled arrangements the most likely Markov state *c*_0_ at low values of {*n*, *I*}. By maximizing coevolutionary information, we were able to solve for the most likely optimized trajectory between *c*_0_ and absorbing state *c*_*k*_, enabling extensive quantification of TP rates across the parameter space. However, limitations in the numerical resolution of the model prevented the computation of finite transition probabilities into states with very low probabilities, thus constraining the location of the absorbing state in such cases. Despite these numerical limitations, which may lead to an underestimation of some predicted values, the model reveals a clear dependence of TP rates on {*M*, α, and σ_0_^2^}. It also demonstrates that the ″sweet spot″ domain of parameters for which TP rates are significant (≥0.5) shrinks considerably as the total number of sequences increases (Figure 4). For *M* ≥ 100, the model converges, effectively ruling out the occurrence of significant rates across the entire parameter space.

### Validation of the Model’s Predictions

To validate the TP predictions generated by the model, we conducted independent simulations of a genetic algorithm (GA) designed to maximize the coevolutionary information on the protein families in Table S1 through a Markov process—where two sequences are swapped per time step. The stochastic dynamics of the simulations closely align with our theoretical framework, i.e., GA simulations maximize the coevolutionary information by adopting the same transition hierarchy assumed in the model *n*_*t*+1_ ∈ {*n*_*t*_ – 1, *n*_*t*_, *n*_*t*_ + 1} (Figure S4). As previously described,^6^ approximately 10 independent GA simulations were run to maximize the coevolutionary information on the protein families, starting from scrambled arrangements. TP rates were averaged over the optimized solutions from the GA simulations and directly compared to the model’s predictions.

All simulation results for the protein families shown in Table S1 are compared with the model’s predictions shown in Figure 4. While reaching scrambled arrangements through the minimization of the coevolutionary signal of native pairs is straightforward, the reverse is not true for large M. The model captures this hysteresis, which is observed in all simulated systems listed in Table S1, further reinforcing the strength of its underlying construction. Except for HK-RR families with 10 or fewer protein copies per genome, all simulated systems with parameters outside the sweet spot domain consistently fail to correctly resolve protein partners (Table S1). On average, HK-RR families with 10 or fewer protein copies per genome achieve significant true-positive (TP) rates after optimization. In contrast, the simulated rates significantly decrease for both paralogous and orthologous families with a greater number of sequences *M*.

### Reassessment of the Statistical Model

Only for a small number of sequences does the model establish a quite generous sweet spot domain for which TP rates are significant. The result then suggests that subjected to optimization of the coevolutionary information, protein families with large *M* may have their partners *A* and *B* properly resolved only at a reduced effective number of sequences. To further explore that possibility, Figures 2, 3, and 4 present in light of eqs 9–14, the reassessment of the statistical model according to the effective number of sequences *n*′ that are paired either with their correct partner or with a similar partner. As detailed in Computational Methods, similar partners of sequences *A* were defined according to a Hamming distance cutoff corresponding to the 20th percentile of the distribution of Hamming distances of sequences *B*, *p* = 0.2 (Figure S5). Consistent with a valid approximation of the parameters θ_*nm*_ ≈ θ_*n*_ (Figures S6 and S7), reassessment of the statistical model modifies primarily the Poisson weights *w*_*n*_′ and as such, the reweighted probability function *w*_*n*′_*f*_*n*′_(*I* | θ_*n*′_) now features a generalized redistribution of probability density along the effective number *n*′. The referred redistribution does not significantly impact the location of the most likely Markov state *c*_0_ in the domain of the reassessed probability density {*n* ′, *I*}. However, it drastically modifies time evolution of the stochastic variable *C* and, consequently, the TP rate attained by the most likely trajectory of the optimization process. By disregarding mismatches made among similar sequences, the reassessed model produces TP rates that are systematically larger than those produced by the original model. The reassessed sweet spot domain {α, σ_0_^2^} is now predicted to be significantly broader for all *M*.

**Figure 5:**
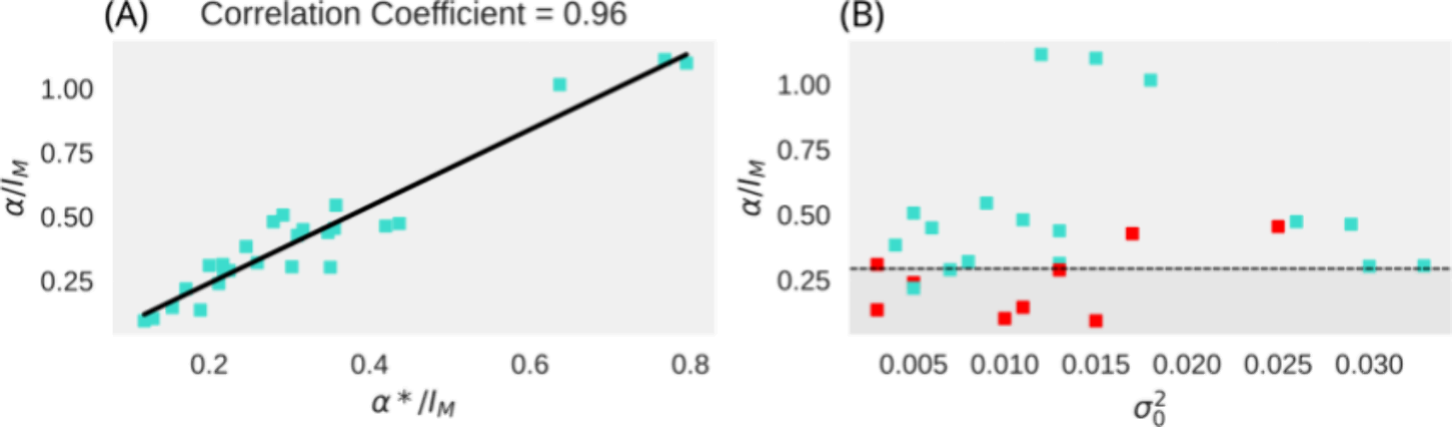
Statistical distinction of optimized solutions with trivial errors. (A) Prediction of α from GA simulations. α* was computed by taking into consideration the optimized coevolutionary information I* (cf. Table S1). (B) Location of protein families across the parameter space {α, σ_0_^2^}. Most systems, where simulated rates improved by more than 25% after reassessing similar sequences (blue), fall within the model’s predicted region of the parameter space, likely containing optimized solutions with trivial errors (dashed line). For each protein family, {*I*_0_, σ_0_^2^} was estimated from ∼5000 randomly generated scrambled arrangements at the fixed number of positions *n* = 0.

While the model’s results can be directly compared to simulation results of the paralogous systems with *M* = 10, 20, and 30, note that the model’s results at *M* = 100 must be extrapolated for proper comparison to simulation results of all orthologous systems with *M* > 100. Extrapolation of the data for large *M* > 100 suggests that optimized solutions with trivial errors must be confined in the region of the parameter space roughly characterized by {α ≥ 15, σ_0_^2^ ≤ 0.5} (cf. Figure 4B). Particularly useful, the result provides us with the potential location that optimized solutions with trivial errors may have across the parameter space, thus making their statistical distinction from other degenerate solutions. Because {α, σ_0_^2^} can be fairly known from GA simulations starting from an ensemble of scrambled arrangements (Figure 5), our quantitative finding then allows for the a *priori* classification of protein families that may have partners *A* and *B* effectively resolved by disregarding trivial errors produced by optimization of the coevolutionary signal. Indeed, most of the parameters for protein families, where the simulated rates can be significantly improved after reassessing trivial errors (≥25%), fall within the predicted domain. This further supports the main conclusions drawn from the model.

## Concluding Remarks

Our research explores the statistical conditions that govern the prediction of protein partners between two interacting families, A and B, by maximizing the coevolutionary information available in their primary sequences. One example where these approaches, as formulated in our study, can be applied is in paralogous families with one or more protein copies per genome. While such predictions are straightforward for prokaryotic genomes—where protein partners are encoded within the same operon and are thus well defined—they become more complex for eukaryotic genomes,^14^ making coevolutionary analyses particularly valuable. Another example involves predicting protein partners between interacting families A and B across independent genomes. Examples are phage proteins and bacterial receptors,^15^ pathogen and host-cell proteins,^16^ neurotoxins,^17^ and ion channels,^18^ among others. The motivation for predicting protein partners across these examples has driven the pioneering work of Bitbol and colleagues,^8^ along with subsequent studies,^5−7^ in which annotated data sets—derived from the P2CS database containing two-component system proteins from all fully sequenced prokaryotic genomes—served as a benchmark for inferring interaction partners from protein sequences.

Ideally, identification of protein partners *A* and *B* would demand the comparative evaluation of the binding free energy by means of docking studies^19^ or advanced atomistic calculations.^20^ However, in practice, this approach is not feasible for many protein pairs, prompting researchers to rely on amino acid sequences to conduct the necessary analyses. The coevolutionary signal corresponds to a small yet important fraction of the total information available in protein sequences,^10^ making them especially suitable for inferring specific partners through fast algorithmic routines.^5−8^ It is worth emphasizing that our modeling study is not intended to provide a method for predicting protein–protein interactions. As such, it differs from previous studies that used coevolutionary signals to predict protein partners based on sequence alignments. Since our statistical model is not an algorithm in itself, it cannot be directly compared to the performance of these introduced methods for identifying protein partners. Rather, our contribution lies in presenting a general modeling framework that rationalizes the statistical behavior of algorithmic predictions of protein partners derived from amino acid sequences.

Here, we investigate specific statistical conditions of the coevolutionary signal that enable algorithmic predictions of protein partners *A* and *B*. More specifically, we investigate a Markov stochastic model of the number of correct protein partners *n* and coevolutionary information *I*. Despite its simple formulation, based on a Poisson mixture of normal distributions, the model seems to retain essential features parametrically described by {*M*, α, σ_0_^2^} that help rationalize the simulation results of protein families. The fact that significant TP rates can be attainable only by simulated systems with parameters inside the sweet spot domain of the model adds support to that conclusion. Particularly important, the predictive power of the model demonstrates that protein families with a large number of sequences *M* ≥ 100 can have their partners effectively resolved only when errors between similar pairs are disregarded. In this case, the reassessed model identifies a specific region of the parameter space {α ≥ 15, σ_0_^2^ ≤ 0.5}, likely containing optimized solutions with trivial errors, including those from protein families where simulated rates can be improved by reassessing similar sequences.

Distinguishing optimized solutions with trivial errors from other degenerate solutions is critical, as it allows for the *a priori* classification of protein families where accurate partner prediction is achievable at the coevolutionary clade level—offering valuable insights for biotechnological applications where potential cognate pairs are unknown. In other words, our model statistically defines the propensity of interacting protein families, in cospeciation or across independent genomes, to have partners effectively resolved at the clade level by disregarding trivial errors produced during the optimization of the coevolutionary signal. Practically, for any given system, the maximization of the coevolutionary signal can be performed by starting with an ensemble of scrambled arrangements. From these scrambled and optimized arrangements, one can compute the system’s parameters {α, σ_0_^2^} to determine whether the optimized solution contains trivial errors—i.e., if it falls within the region of the parameter space roughly characterized by {α ≥ 15, σ_0_^2^ ≤ 0.5}.

To the best of our knowledge, this is the first study that attempts to investigate the statistical production of TP rates in coevolutionary approaches from a modeling perspective. Although our model relies on the maximization of coevolutionary information, it is worth mentioning that its structure is general and should help explore the production of TP rates according to other heuristics, such as the Metropolis algorithm. We thus believe the results are of broad interest, as the parameters {*M*, α, σ_0_^2^} appear to be critical for coevolutionary approaches in general. We therefore anticipate that the novel theoretical insights presented here might provide relevant information for future studies and should contribute to advancing our knowledge in the field.

## Data Availability

All numerical calculations of the statistical model can be reproduced following the tutorial made available for download at GITHUB https://github.com/jafiorote/ga_error_sources. A complete collection of scripts for running Genetic Algorithm simulations and performing parameter calculations—including *I*′, *I**, *I*_0_, σ_0_^2^ and TP rate—for both examples of orthologous and paralogous protein families is available for download from the ZENODO repository https://zenodo.org/records/14624294.

## Supporting Information Available

(Figure S1) Distribution of the coevolutionary information; (Figure S2) dependence of the Poisson weights *w*_*n*_ with the number of correct partners *n*; (Figure S3) probability density of coevolutionary information *f*_*n*_(*I* | θ_*n*_) as a function of the number of correct partners *n*; (Figure S4) GA simulations; (Figure S5) distribution of Hamming distances of sequences B; (Figure S6) distribution of similar partners as a function of the number of fixed positions; (Figure S7) approximation of the distribution parameters θ_*nm*_ ≈ θ_*n*_ as a function of the number of fixed positions; and (Table S1) protein families considered in the study (PDF)
