# Supplementary material for: Investigating Statistical Conditions of Coevolutionary Signals that Enable Algorithmic Predictions of Protein Partners
Source: J Chem Inf Model. 2025 Apr 15;65(8):4107–15. doi: 10.1021/acs.jcim.5c00052 (PMC12042258; doi:10.1021/acs.jcim.5c00052)
Supplement: Supplementary file 1 — ci5c00052_si_001.pdf [file ci5c00052_si_001.pdf]

Supporting Information

Investigating Statistical Conditions of Coevolutionary Signals that Enable Algorithmic Predictions of Protein Partners

José Fiorote,[1] João Alves,[1] Letícia Stock[2] and Werner Treptow[1]

[1]Laboratório de Biologia Teórica e Computacional (LBTC), Universidade de Brasília DF, Brasil

[2]Ben May Department for Cancer Research, University of Chicago, Chicago, IL 60637, USA

Author contributions. JF and JA contributed equally to this work.

To whom correspondence may be addressed. E-mail. treptow@unb.br

SUPPLEMENTARY RESULTS, FIGURES AND TABLES

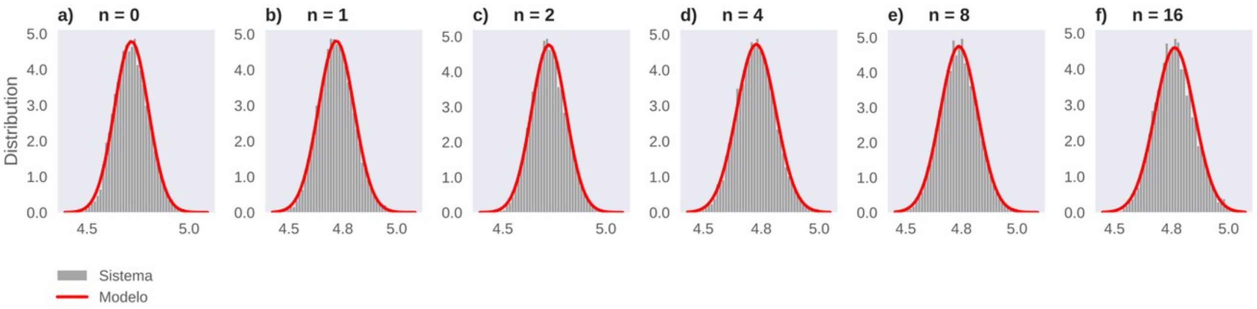

Figure S1. Distribution of the coevolutionary information. Shown is the distribution of coevolutionary information for the protein family 1BXR, consisting of 1004 sequences. Each distribution was generated by considering approximately 10,000 random arrangements at a fixed number of positions, n. Based on eq. [4] in the main text, the distributions are well described by Gaussian models, with parameters corresponding to the parameters of 1BXR shown in Table-S1.

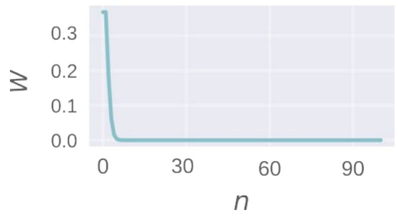

Figure S2. Dependence of the Poisson weights w_n with the number of correct partners n.

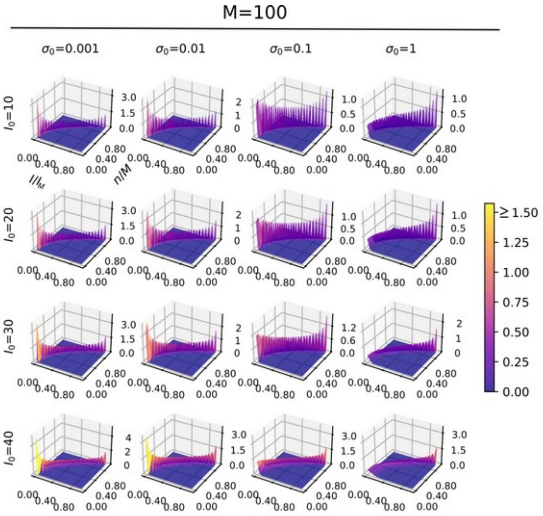

Figure S3. Probability density of coevolutionary information $f_n(I|\boldsymbol{\theta}_n)$ as a function of the number of correct partners n. The probability density is shown for a fixed number of sequences $M=100$.

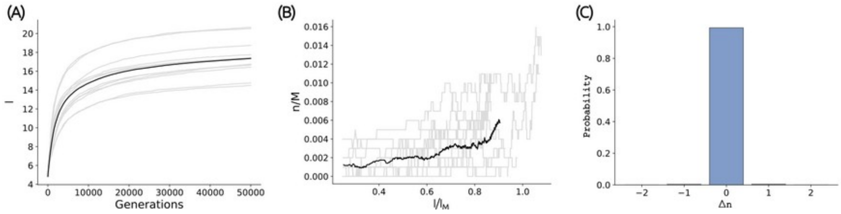

Figure S4. GA simulations. (**A**) Maximization of the coevolutionary information I throughout ~10 independent GA simulations of the protein system 1BXR with $M=1004$ sequences (Table-S1). The average trajectory is shown in black. (**B**) Time evolution of GA simulations across the domain $\{n, I\}$. The average trajectory is shown in black. (**C**) Distribution of transitions Δn involved in the swapping of two sequences per time step of the GA simulations. Best ranked solutions (elite) were considered for computation of Δn. Note that GA simulations maximizes the coevolutionary information by adopting the same transition hierarchy assumed in the model, *i.e.*, $n_{t+1} \in \{n_t - 1, n_t, n_t + 1\}$.

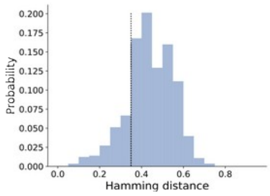

Figure S5. Distribution of Hamming distances of sequences B. Shown is the Hamming distribution for the protein system 1BXR. Dashed line indicates the 20th percentile of the Hamming distribution considered as the cutoff for trivial mismatch discounting ($p=0.2$).

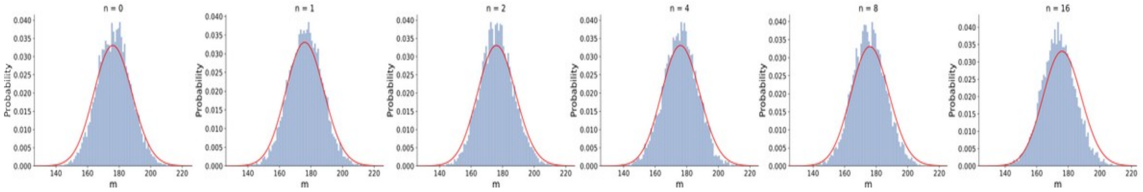

Figure S6. Distribution of similar partners as a function of the number of fixed positions. Distributions are shown for the protein system 1BXR with $M=1004$ sequences. Random arrangements were generated at a fixed number of positions n and subsequently distributed according to the number of similar partners m, defined at constant probability $p=0.2$ (*cf.* Fig. S5). Data is consistent with the Binomial distribution B_m^{M-n} devised in eq. [13] (red line).

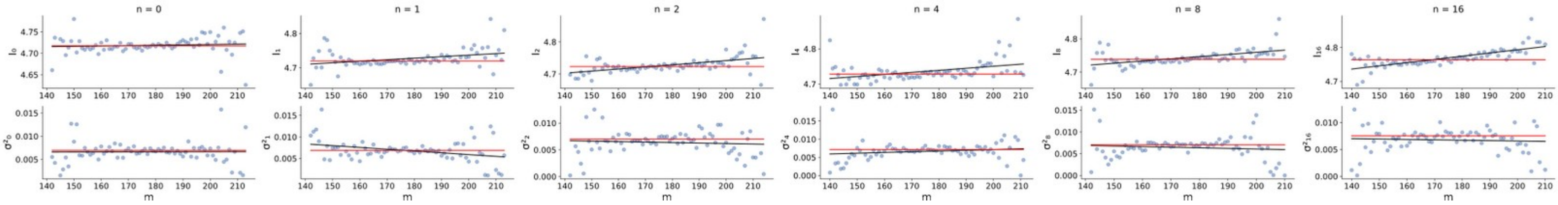

Figure S7. Approximation of the distribution parameters $\boldsymbol{\theta}_{nm} \approx \boldsymbol{\theta}_n$ as a function of the number of fixed positions. From Fig. S5, shown is the average I_{nm} and variance σ^2_{nm} of the mutual information in each subset m of the distribution $\boldsymbol{\theta}_{nm}$ with Binomial probability $B_m^{M-n} > 0$. Linear regression of the data (black line) superposes well to the same estimates of the source distribution $\boldsymbol{\theta}_n$ (red line), especially across the most likely values of m. The analysis thus indicates that $\boldsymbol{\theta}_{nm} \approx \boldsymbol{\theta}_n$ is a valid approximation across small values of n for which Poisson weights are relevant $w_n > 0$.

Table-S1. Protein families considered in the study#

	Systems	M	I'	I_0	σ_0^2	α/I_M	I^*	TP (model)	TP_e (model)
Paralogous	**HK-RR (average over 16 species)**	10	15.913±9.960	14.861 ±11.110	0.263±0.010	0.021	15.790±10.050	0.500 (0.100±0.075)	0.687 (0.350±0.106)
Paralogous	**HK-RR (average over 4 species)**	20	16.592±9.660	14.537 ±6.902	0.195±0.001	0.041	16.026±5.362	0.3125 (0.012±0.021)	0.500 (0.337±0.064)
Paralogous	**HK-RR (average over 6 species)**	30	14.319±8.255	12.200 ±5.965	0.122±0.000	0.042	13.791±6.070	0.205 (0.016±0.016)	0.583 (0.333±0.083)
orthologous	**1B70_AB**	1108	35.671	11.761	0.011	0.480	25.989	0.004 (0.04)	0.295 (0.980)
orthologous	**1BXR_AB**	1004	19.179	4.717	0.007	0.289	15.965	0.006 (0.13)	0.312 (0.900)
orthologous	**1EFP_AB**	1347	65.050	14.305	0.018	1.015	46.174	0.006 (0.02)	0.362 (1.000)
orthologous	**1EP3_AB**	552	22.945	7.762	0.033	0.304	22.916	0.009 (0.03)	0.373 (0.900)
orthologous	**1I1Q_AB**	1204	9.752	3.014	0.003	0.135	12.449	0.001 (0.63)	0.200 (0.720)
orthologous	**1QOP_AB**	1155	14.741	2.773	0.005	0.239	13.380	0.002 (0.82)	0.215 (0.860)
orthologous	**1RM6_AB**	1604	19.673	4.212	0.003	0.309	14.228	0.001 (0.07)	0.158 (0.900)
orthologous	**1RM6_AC**	1534	27.484	5.020	0.006	0.449	20.867	0.003 (0.04)	0.267 (0.970)
orthologous	**1RM6_BC**	1481	24.015	4.863	0.004	0.383	17.185	0.003 (0.04)	0.316 (0.940)
orthologous	**1TYG_AB**	746	15.397	4.515	0.005	0.218	13.074	0.003 (0.79)	0.272 (0.840)
orthologous	**1W85_AB**	1537	31.162	5.879	0.005	0.506	20.469	0.002 (0.03)	0.315 (0.980)
orthologous	**1ZUN_AB**	649	23.727	8.612	0.030	0.302	26.153	0.011 (0.04)	0.390 (0.900)
orthologous	**2D1P_BC**	216	15.541	8.268	0.011	0.145	15.948	0.010 (0.66)	0.203 (0.740)
orthologous	**2NU9_AB**	798	29.521	5.879	0.026	0.473	27.721	0.011 (0.04)	0.315 (0.980)
orthologous	**2VPZ_AB**	676	36.305	13.581	0.025	0.454	31.400	0.003 (0.04)	0.166 (0.970)
orthologous	**2WDQ_CD**	221	7.864	3.192	0.015	0.093	9.145	0.018 (0.36)	0.194 (0.590)
orthologous	**2Y69_AB**	1484	65.845	10.219	0.012	1.113	48.620	0.006 (0.01)	0.500 (1.000)
orthologous	**2Y69_AC**	863	32.533	9.405	0.029	0.463	30.417	0.006 (0.03)	0.388 (0.980)
orthologous	**3G5O_AB**	904	32.880	10.978	0.013	0.438	28.374	0.004 (0.04)	0.313 (0.970)
orthologous	**3IP4_AB**	782	7.168	2.081	0.010	0.102	8.578	0.005 (0.42)	0.160 (0.620)
orthologous	**3IP4_AC**	879	22.003	6.375	0.013	0.313	17.204	0.009 (0.09)	0.336 (0.910)
orthologous	**3IP4_BC**	689	21.928	7.552	0.013	0.288	18.430	0.004 (0.08)	0.248 (0.900)
orthologous	**3MML_AB**	1067	34.283	7.071	0.009	0.544	24.969	0.004 (0.03)	0.390 (0.990)
orthologous	**3OAA_HG**	886	34.312	12.954	0.017	0.427	28.435	0.002 (0.05)	0.206 (0.970)
orthologous	**3PNL_AB**	902	20.740	4.756	0.008	0.320	17.766	0.004 (0.09)	0.303 (0.910)
orthologous	**3RRL_AB**	1330	62.525	7.554	0.015	1.099	47.312	0.006 (0.01)	0.340 (1.000)

M is the total number of primary sequences. I' is the coevolutionary information of the native arrangement, measured in *nats*. $\{I_0, \sigma_0^2\}$ are scrambled parameters determined by averaging over random arrangements with $n=0$. $\alpha = I' - I_0$ is the information gap. I^* is the maximized coevolutionary information averaged over ~10 independent GA simulations. TP is the true-positive rate averaged over optimized solutions obtained from GA simulations. TP_e is the true-positive rate averaged over optimized solutions after the reassessment of mismatches among similar sequences ($p=0.2$). Model's predictions for both rates are shown in parentheses. Orthologous families having simulated rates improved above 25% after the reassessment of trivial errors are highlighted in light blue. While the model's results can be directly compared to simulation results of the paralogous systems with M=10, 20, and 30, note that the model's results at M=100 must be extrapolated for proper comparison to simulation results of all orthologous systems with M>100.
